# Interactions between migraine and tension-type headache and alcohol drinking, alcohol flushing, and hangover in Japanese

**DOI:** 10.1007/s10194-011-0413-6

**Published:** 2012-01-11

**Authors:** Masako Yokoyama, Norihiro Suzuki, Tetsuji Yokoyama, Akira Yokoyama, Kazuo Funazu, Toshihiko Shimizu, Mamoru Shibata

**Affiliations:** 1 Keio Research Consortium for Migraine Epidemiology, Tokyo, Japan; 2 Mitsukoshi Health and Welfare Foundation, 1-24-1 Nishishinjuku, Shinjuku-ku, Tokyo, 169-0023 Japan; 3 Department of Health Promotion, National Institute of Public Health, Saitama, Japan; 4 National Hospital Organization Kurihama Alcoholism Center, Kanagawa, Japan

**Keywords:** Alcohol, Alcohol flushing, Aldehyde dehydrogenase-2, Hangover, Migraine, Tension-type headache

## Abstract

The aim of the study was to investigate associations between headache types and alcohol drinking, alcohol flushing, and hangover. Alcohol consumption is inhibited by the presence of inactive aldehyde dehydrogenase-2 (ALDH2) whose carriers are susceptible to alcohol flushing and hangovers. We conducted a cross-sectional study of the 2,577 subjects (men/women: 1,018/1,559) who reported having ever experienced headaches unrelated to common colds and alcohol hangovers among 5,408 (2,778/2,630) Tokyo health checkup examinees. We used a questionnaire inquiring about current and past facial flushing after drinking a glass of beer which identifies the presence of inactive ALDH2 with a sensitivity and specificity of approximately 90%. Based on ICHD-II criteria migraine was diagnosed in 419 (75/344) subjects, and tension-type headache (TTH) in 613 (249/364). We classified the headaches of the remaining 1,545 (694/851) of headaches sufferers into the category “other headaches (OH)”. The migraineurs drank alcohol less frequently than the subjects with TTH among current/past alcohol flushers and than the subjects with OH regardless of flushing category. No such difference in drinking frequency was observed between TTH and OH. Current/past flushers drank alcohol less frequently than never flushers, and the likelihood that male migraineurs would avoid alcohol drinking than men with TTH or OH was stronger among current/past flushers than among never flushers. Flushers and women were more susceptible to hangover than never flushers and men, respectively, regardless of headache type. Among never flushers, women with migraine were more susceptible to hangover than women with OH. The difference in alcohol sensitivity may partly explain less alcohol consumption by migraineurs.

## Introduction

Our previous cross-sectional survey of 12,988 subjects undergoing health checkups at a Tokyo clinic showed that headache sufferers of both genders reported less alcohol consumption [1]. The inverse dose–response relationship between alcohol consumption and headaches is consistent with the results of other large population-based cross-sectional surveys [2–6]. A mutant allele encoding an inactive subunit of aldehyde dehydrogenase-2 (ALDH2; rs671) was carried by Han Chinese as they spread throughout East Asia, and it is not found in Caucasians or Africans [7]. Alcohol consumption by Japanese is strongly inhibited by the presence of inactive ALDH2, because ALDH2-deficient individuals are more susceptible to alcohol flushing responses [8, 9] and hangovers [10, 11]. Headache is a major symptom of flushing responses [9] and hangovers [11]. Consumption of alcoholic beverages has been reported to be a factor that aggravates migraine [12–14] and cluster headaches [15]. Individuals with migraine are at higher risk of delayed alcohol-induced headache, previously named hangover headache, than people without migraine and phenotypically both types of headache have similar clinical features [16]. Headaches associated with alcohol flushing and hangover are at least in part mediated by acetaldehyde production [8–11]. The susceptibility of Japanese headache sufferers to alcohol-associated headaches may vary with the type of headache and their ALDH2 genotype. A questionnaire inquiring about current and past facial flushing after drinking a glass (≈180 ml) of beer is capable of identifying the presence of inactive ALDH2 with a sensitivity and specificity of approximately 90% in both genders [17, 18]. The question about past facial flushing is important, because some individuals become tolerant to the facial flushing effect as a result of habitual drinking [17]. Alcohol flushing is such a memorable experience that abstainers based on their past experience replied about their current status probably because 96–98% of the inactive ALDH2 homozygotes, who were generally nondrinkers, classified themselves as current flushers [9, 17].

The present study was a large cross-sectional study which was conducted in a Tokyo clinic by using a headache questionnaire designed to diagnose headache type according to the ICHD-II criteria [19] and a questionnaire regarding drinking behavior, alcohol flushing, and hangover. The aim of the study was to investigate associations between headache types and alcohol drinking, alcohol flushing, and hangover.

## Subjects and methods

We conducted a cross-sectional study of 5,408 subjects (men/women: 2,778/2,630) undergoing health checkups at the Mitsukoshi clinic in Tokyo between September 2008 and March 2009. All the subjects worked in Tokyo and had been registered at the clinic for annual health checkups. They were routinely asked to fill out a self-administered headache questionnaire designed to diagnose headache type according to the ICHD-II criteria, and the questionnaire inquired about age (19 years or under, 20–24 years, 25–29 years, 30–34 years, 35–39 years, 40–44 years, 45–49 years, 50–54 years, 55–59 years, or 60 years or older), sex, frequency of headache (once, 2–4 episodes, 5–9 episodes, 10 or more episodes, or numerous episodes), average frequency of headache during the preceding year (less than 1 episode/month, 1–14 episodes/month, or 15 or more episodes/month), headache duration when untreated (less than 30 min, between 30 min and 4 h, more than 4 h but no more than 1 day, 2–3 days, 4–7 days, or more), location of headache (unilateral and/or bilateral), characteristics (pulsating and/or pressing/tightening quality), pain intensity (mild, moderate, or severe), aggravation by routine physical activity (e.g., walking or climbing stairs), associated symptoms (nausea, vomiting, photophobia, and/or phonophobia), and aura (flickering lights, spots or lines, and/or homonymous visual symptom). Migraine was diagnosed if headache attacks fulfilled the diagnostic criteria for migraine without aura. Although we asked about the occurrence of visual symptoms before the headache, since it was difficult to determine whether such symptoms were truly indicative of focal cerebral dysfunction as defined by the ICHD-II, in our study both migraine without aura and migraine with aura were classified simply as migraine. Tension-type headache was also diagnosed according to the diagnostic criteria of the ICHD-II. If headache fulfilled the diagnostic criteria for infrequent episodic tension-type headache, frequent episodic tension-type headache, or chronic tension-type headache, it was diagnosed as tension-type headache. Headache that did not fulfill the diagnostic criteria for migraine or tension-type headache, including probable migraine and probable tension-type headache, was classified into the category of other headache.

The drinking questionnaire asked only the subjects who reported having ever experienced headaches other than headaches related to common colds and alcohol hangovers to report their frequency of alcohol consumption as none, occasional, or habitual (drinking on 1 or more days/week). Habitual drinkers were asked to report the number of day(s)/week they usually consumed alcohol and the usual amount of alcohol consumed in the form of as unit(s)/day (1 unit = 22 g ethanol, the ethanol content of one serving of sake). Each subject was asked to fill out a simple questionnaire concerning alcohol flushing [17] that asked (a) Do you have a tendency to develop facial flushing immediately after drinking a glass (≈180 ml) of beer (yes, no, or unknown)? (b) Did you have a tendency to develop facial flushing immediately after drinking a glass of beer in the first 1 or 2 years after you started drinking (yes, no, or unknown)? The designation “current flushers” was applied to individuals who answered “yes” to question (a); “past flushers,” to those who answered “no” or “unknown” to question (a) and “yes” to question (b). The remaining subjects were classified as “never flushers.” We also asked the subjects to specify the usual amount of alcohol consumed that was followed by a hangover (<1, 1–1.9, 2–2.9, 3–3.9, 4–4.9, 5–5.9, ≥6 units, or unknown).

This study was conducted in accordance with the Ethical Guidelines for Epidemiological Research in Japan and reviewed and approved by the Ethics Committee of the Mitsukoshi Health and Welfare Foundation. Informed consent was obtained from each subject.

## Statistical analysis

Data were summarized as percentage values and the Chi-squared test and Cochran–Mantel–Haenszel test for trend adjusted for age and sex were used for comparisons between groups. A multiple logistic regression model was used to calculate age-adjusted odds ratios (ORs) and the 95% confidence interval (CI). A two-sided *p* value <0.05 was considered statistically significant. All statistical analyses were performed using SAS software (version 9.1, SAS Institute, Cary, NC).

## Results

Of the 5,408 (2,778/2,630) Tokyo health checkup examinees, 2,577 [men/women: 1,018 (36.6%)/1,559 (59.3%)] reported having ever experienced headaches other than headaches related to common colds and alcohol hangovers, and answered the questions related to drinking behavior, alcohol flushing, and hangover. Among those who replied that they had ever experienced headaches, migraine was diagnosed according to the ICHD-II criteria in 419 [men/women: 75 (2.7%)/344 (13.1%)] and tension-type headache in 613 [249 (9.0%)/364 (13.8%)]. We classified the headaches of the remaining 1,545 [694 (25.0%)/851 (32.4%)] of headaches sufferers into the category “other headaches” to use them as controls, and their headaches tended to be less frequent and milder than the migraine and tension-type headaches. The age distribution significantly differed by headache type, and 30–49 age-brackets were more frequent in the subjects with migraine and tension-type headache than the subjects with “other headaches” (Table 1). To decrease the confounding effect of age, a statistical adjustment for age was made in the subsequent analyses.

**Table 1:** Age-distribution and headache types

	*N*	Age (years)						*P* ^a^
		<20 (%)	20–29 (%)	30–39 (%)	40-49 (%)	50–59 (%)	60+ (%)	
Men								
Migraine	75	0.0	9.3	38.7	28.0	17.3	6.7	0.004
Tension-type headache	249	0.0	10.0	24.1	32.9	27.7	5.2	
Other headaches	694	0.1	9.4	21.6	24.6	37.2	7.1	
Total	1,018	0.1	9.5	23.5	26.9	33.4	6.6	
Women								
Migraine	344	0.3	7.6	43.0	36.6	11.3	1.2	<0.0001
Tension-type headache	364	0.0	11.3	40.1	37.9	9.9	0.8	
Other headaches	851	0.4	13.8	38.0	28.7	16.7	2.6	
Total	1,559	0.3	11.8	39.6	32.6	13.9	1.9	
Both sexes								
Migraine	419	0.2	7.9	42.2	35.1	12.4	2.2	<0.0001
Tension-type headache	613	0.0	10.8	33.6	35.9	17.1	2.6	
Other headaches	1,545	0.3	11.8	30.6	26.9	25.9	4.6	
Total	2577	0.2	10.9	33.2	30.3	21.6	3.7	

^a^Chi-squared test

The replies to the flushing questionnaire revealed that 45.6% of the 2,577 headache sufferers (men/women: 48.3%/43.8%) were current or past flushers, and they were predicted to have inactive ALDH2. Among the headache sufferers, regardless of headache type, current/past flushers drank alcohol less frequently than never flushers (Table 2). The subjects with migraine drank alcohol less frequently than the subjects with tension-type headache among current/past flushers and than the subjects with “other headaches” regardless of flushing category. According to gender, men with migraine drank less frequently than men with tension-type headache or “other headaches” among current/former flushers, and women with migraine drank less frequently than women with “other headaches” among never flushers. No such difference in drinking frequency was observed between the subjects with tension-type headache and the subjects with “other headaches”. When drinking frequency was classified as none, occasional, 1–3 days/week, 4–6 days/week, or every day and the subjects with “other headaches” were used as controls, the decreasing trend in migraine risk according to category of drinking frequency was significantly more marked in men who were current/past flushers than in men who were never flushers [age-adjusted ORs (95% CIs) per +1 category increment in drinking frequency 0.45 (0.28–0.74) and 0.92 (0.61–1.38), respectively; *p* = 0.039 for difference in OR; Table 3]. No decreasing trend in risk of tension-type headache was observed in any flushing category regardless of gender. When men and women were combined, the decreasing trend in risk according to category of drinking frequency was significant only in migraineurs who were current/past flushers [0.63 (0.49–0.81)]. When the subjects with tension-type headache were used as the reference group, the decreasing trend in migraine risk according to category of drinking frequency was significant in men who were current/past flushers [0.55 (0.33–0.92)], women who were never flushers [0.74 (0.56–0.97)], and men and women who were current/past flushers [0.71 (0.54–0.93)], and was significantly more marked in men who were current/past flushers than in men who were never flushers (*p* = 0.0498 for difference in OR; Table 4).

**Table 2:** Relationships between drinking frequency and headache type according to alcohol flushing

		Never flushers						Current or past flushers				
	*N*	Frequency of alcohol drinking			*P* for homogeneity/trend^a^			Frequency of alcohol drinking			*P* for homogeneity/trend^a^	
		Habitual (%)	Occasional (%)	None (%)	Versus other headaches	Versus tension-type headache	*N*	Habitual (%)	Occasional (%)	None (%)	Versus other headaches	Versus tension-type headache
Men												
Migraine	36	66.7	25.0	8.3	0.167/0.071	0.433/0.227	39	15.4	23.1	61.5	0.001/<0.001	0.011/0.003
Tension-type headache	118	75.4	21.2	3.4	0.114/0.296	–	131	37.4	22.1	40.5	0.285/0.113	–
Other headaches	372	82.5	12.9	4.6	–	–	322	45.0	22.4	32.6	–	–
Women												
Migraine	186	36.0	42.5	21.5	0.037/0.044	0.485/0.233	158	12.7	30.4	57.0	0.191/0.072	0.348/0.273
Tension-type headache	214	42.5	40.7	16.8	0.320/0.782	–	150	19.3	28.7	52.0	0.441/0.724	–
Other headaches	477	45.1	35.4	19.5	–	–	374	17.6	35.6	46.8	–	–
Both sexes												
Migraine	222	41.0	39.6	19.4	0.009/0.012	0.295/0.119	197	13.2	28.9	57.9	0.002/<0.001	0.015/0.008
Tension-type headache	332	54.2	33.7	12.0	0.065/0.463	–	281	27.8	25.6	46.6	0.266/0.160	–
Other headaches	849	61.5	25.6	13.0	–	–	696	30.3	29.5	40.2	–	–

Among the headache sufferers, regardless of headache type, current/past flushers drank alcohol less frequently (*p* for trend <0.0001) than never flushers by Cochran–Mantel–Haenszel test for trend

^a^Cochran–Mantel–Haenszel test for homogeneity/trend adjusted for age (and for sex when both sexes were combined)

**Table 3:** Drinking frequency and odds ratio of migraine and tension-type headache in comparison with other headaches according to alcohol flushing

	Age-adjusted OR (95% CI) in comparison with other headaches						
	Frequency of alcohol drinking				*P* for trend	+1 category of drinking frequency	*P* for difference in OR
	None	Sometimes/1–3 days/week	4–6 days/week	Every day			
Men							
Migraine							
Never flushers	1 (ref.)	0.71 (0.18–2.83)	0.63 (0.15–2.70)	0.67 (0.16–2.86)	0.680	0.92 (0.61–1.38)	0.039
Current/past flushers	1 (ref.)	0.51 (0.25–1.06)	0.11 (0.02–0.87)	0.12 (0.02–0.92)	0.001	0.45 (0.28–0.74)	
Tension-type headache							
Never flushers	1 (ref.)	2.04 (0.65–6.41)	1.57 (0.49–5.06)	0.99 (0.30–3.25)	0.082	0.80 (0.62–1.03)	0.810
Current/past flushers	1 (ref.)	0.83 (0.51–1.33)	0.40 (0.17–0.91)	0.71 (0.36–1.43)	0.092	0.83 (0.66–1.03)	
Women							
Migraine							
Never flushers	1 (ref.)	1.04 (0.67–1.61)	0.41 (0.19–0.85)	1.01 (0.44–2.31)	0.185	0.85 (0.67–1.08)	0.532
Current/past flushers	1 (ref.)	0.68 (0.46–1.02)	0.91 (0.30–2.70)	0.39 (0.08–1.81)	0.060	0.75 (0.56–1.01)	
Tension-type headache							
Never flushers	1 (ref.)	1.18 (0.75–1.85)	1.13 (0.62–2.05)	1.99 (0.95–4.16)	0.143	1.18 (0.95–1.46)	0.317
Current/past flushers	1 (ref.)	0.75 (0.50–1.14)	1.24 (0.44–3.48)	1.35 (0.48–3.86)	0.863	0.98 (0.74–1.29)	
Both sexes							
Migraine							
Never flushers	1 (ref.)	0.97 (0.64–1.48)	0.49 (0.27–0.91)	0.76 (0.40–1.47)	0.096	0.84 (0.68–1.03)	0.104
Current/past flushers	1 (ref.)	0.64 (0.45–0.90)	0.43 (0.18–1.06)	0.23 (0.07–0.76)	<0.001	0.63 (0.49–0.81)	
Tension-type headache							
Never flushers	1 (ref.)	1.32 (0.87–1.99)	1.21 (0.74–1.98)	1.06 (0.61–1.82)	0.942	0.99 (0.84–1.17)	0.724
Current/past flushers	1 (ref.)	0.79 (0.58–1.07)	0.58 (0.31–1.09)	0.85 (0.48–1.50)	0.142	0.88 (0.74–1.04)	

ORs were calculated by the multiple logistic regression model adjusted for age (and sex)

*OR* odds ratio, *CI* confidence interval

**Table 4:** Drinking frequency and odds ratio of migraine in comparison with tension-type headache according to alcohol flushing

	Age-adjusted OR (95% CI) in comparison with tension-type headache						
	Frequency of alcohol drinking				*P* for trend	+1 category of drinking frequency	*P* for difference in OR
	None	Sometimes/1–3 days/week	4–6 days/week	Every day			
Men							
Migraine							
Never flushers	1 (ref.)	0.24 (0.04–1.32)	0.29 (0.05–1.62)	0.51 (0.09–2.83)	0.646	1.12 (0.70–1.80)	0.0498
Current/past flushers	1 (ref.)	0.57 (0.25–1.28)	0.26 (0.03–2.24)	0.16 (0.02–1.31)	0.022	0.55 (0.33–0.92)	
Women							
Migraine							
Never flushers	1 (ref.)	0.89 (0.52–1.51)	0.38 (0.16–0.88)	0.52 (0.21–1.27)	0.027	0.74 (0.56–0.97)	0.754
Current/past flushers	1 (ref.)	0.92 (0.57–1.48)	0.72 (0.21–2.48)	0.29 (0.06–1.48)	0.181	0.80 (0.57–1.11)	
Both sexes							
Migraine							
Never flushers	1 (ref.)	0.77 (0.46–1.27)	0.42 (0.21–0.85)	0.63 (0.30–1.31)	0.060	0.80 (0.63–1.01)	0.400
Current/past flushers	1 (ref.)	0.81 (0.54–1.23)	0.56 (0.20–1.54)	0.24 (0.07–0.84)	0.013	0.71 (0.54–0.93)	

ORs were calculated by the multiple logistic regression model adjusted for age (and sex)

*OR* odds ratio, *CI* confidence interval

**Fig. 1:**
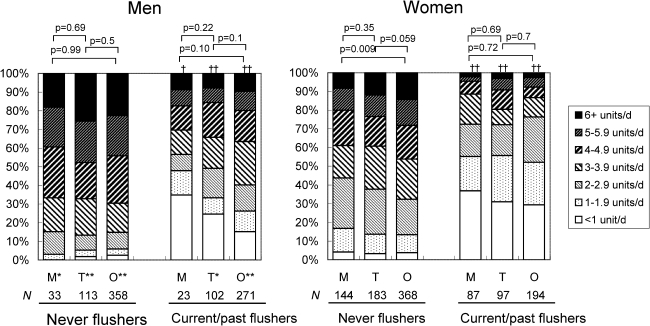
Relationships between the amount of alcohol consumption followed by a hangover and headache type according to alcohol flushing and gender. *M* migraine, *T* tension-type headache, and *O* “other headaches”. Current or past flushers and women were more susceptible to hangovers than never flushers and men, respectively, regardless of headache type. Among the never flushers, women with migraine were more susceptible to hangovers than women with “other headaches”. The statistical analysis was performed by the Cochran–Mantel–Haenszel test for trend adjusted for age. ^†^ *p* = 0.0005, ^††^ *p* < 0.0001 in comparison with never flushers; *p < 0.01, **p < 0.0001 in comparison with women

Among the subjects who specified the usual amount of alcohol consumed that was followed by a hangover, the amount of drinking reported to lead to hangover was significantly less among current/past flushers than never flushers regardless of gender or type of headache, and was significantly less among women than men regardless of alcohol flushing or type of headache (Fig. 1). Among the never-flushers, the women with migraine were significantly more susceptible to hangover (*p* = 0.009) than the women with “other headaches”. Hangover susceptibility did not differ according the type of headache among the men or women who were current/past flushers.

## Discussion

The results of this large cross-sectional survey of headaches in a Japanese population in Tokyo showed that migraineurs drank alcohol less frequently than individuals with tension-type headache or other less-frequent and milder headaches. The proportion of the subjects who had migraine was 2.7% of the men and 13.1% of the women, similar to the proportion of 2.3–3.6% of men and 9.1–12.9% of women reported in earlier Japanese population-based studies [20, 21]. Interactions between the results of the flushing questionnaire, drinking frequency, and headache differed according to headache type. Current/past flushers drank alcohol less frequently than never flushers among the headache sufferers regardless of headache type, but the likelihood that male migraineurs would avoid alcohol drinking than men with tension-type headache or “other headaches” was stronger among current/past flushers than among never flushers. Any significant differences in drinking frequency were not observed between subjects with tension-type headache and subjects with “other headaches”. Possible mechanisms by which alcohol induces headache [22, 23] include a vasodilatory effect on intracranial vasculature [12], alteration of cytokine pathways [24], hormonal disturbance [23], the headache provoking effects of congeners [12], and acetaldehyde-mediated mechanisms [9–11]. Male migraineurs with alcohol flushing may be more susceptible to severe alcohol-induced headache than non-migrainous headache sufferers with alcohol flushing, and that may make them more likely to avoid alcohol. This speculation is also supported by a European study that demonstrated a higher frequency of alcohol dehydrogenase-1B (ADH1B, previously called ADH2) His allele (rs1229984) in migraineurs who reported triggering of migraine attacks by alcohol than in migraineurs who reported no effect of alcohol [14]. The ADH1B polymorphism is a major functional polymorphism for alcohol metabolism among European populations, in which the prevalence of the inactive ALDH2 is extremely low [7], and Europeans with the ADH1B His allele tend to report alcohol flushing and to be prevented from drinking heavily because of their faster acetaldehyde production capacity [25].

The results of this study showed that the amount of alcohol consumption that was followed by a hangover was significantly lower among subjects who were current/past flushers than among those who were never flushers regardless of headache type. This finding is in good agreement with the results of earlier studies which demonstrated a positive association between inactive ALDH2 and hangover susceptibility and suggested a major causal role of acetaldehyde in the development of hangovers [10, 11]. The present results also demonstrated that regardless of flushing category or headache type, women are more vulnerable to hangover than men and that among never flushers women with migraine are more susceptible to hangover than women with “other headaches”. In our earlier studies [11, 26], the more the subjects drank, the more likely they were to develop tolerance to hangovers and hangover headaches. Since migraineurs, especially female migraineurs, tended to consume alcohol less frequently in the present study, they may have had less opportunity to develop tolerance to hangovers.

Our study had several potential limitations. The first potential limitation was that it was a cross-sectional survey based on the results of the questionnaire about alcohol flushing and the possible causation is highly speculative. In addition, we did not perform ALDH2 genotyping, although when current or former flushers classified by the flushing questionnaire were assumed to have inactive ALDH2, both the sensitivity and specificity are approximately 90% [17, 18]. The second potential limitation was that we used the group with “other headaches”, not a group with “no headaches”, as the reference group, because according the study design only the subjects who reported having ever experienced headaches were asked to fill out the alcohol-related questionnaire. The group with “other headaches” that did not fulfill the diagnostic criteria for migraine or tension-type headache included probable migraine and probable tension-type headache. The third potential limitation was that we did not ask the subjects whether or how often their headache attacks were precipitated by drinking alcohol. The percentages of headache patients reporting alcohol as a trigger have been reported to be 17–42% [12]. Individual difference in susceptibility to alcohol flushing and hangover may be associated with individual difference in the trigger effect of alcohol. Another limitation was the difficulty of precisely defining hangover headache. Differentiating between hangover headache or delayed alcohol-induced headache and usual headache or migraine triggered by alcohol is sometimes difficult in non-migrainous headache and migraine subjects [27]. Alcohol hangover is a set of unpleasant symptoms that includes headache, nausea, anorexia, fatigue, and diarrhea in the morning after alcohol intake [11, 23], and some of these symptoms are very common in a migraine attack.

In conclusion, Japanese migraineurs drink alcohol less frequently than subjects with tension-type headache or other less-frequent and milder headaches. Interactions between alcohol drinking, alcohol flushing and hangover susceptibility, and headache differ according to the type of headache and gender and that may partly explain why headache sufferers, especially migraineurs, tend to avoid drinking alcohol.
